# Supplementary material for: Spatiotemporal dynamics of maximal and minimal EEG spectral power
Source: PLoS One. 2021 Jul 20;16(7):e0253813. doi: 10.1371/journal.pone.0253813 (PMC8291701; doi:10.1371/journal.pone.0253813)
Supplement: Attachment — Submitted filename: MencelogluEtAl_response_to_rev2.pdf [file pone.0253813.s001.pdf]

Satoru Suzuki
Professor

Department of Psychology and Interdepartmental Neuroscience Program
2029 Sheridan Road
Evanston, IL 60208-2710 USA

Telephone: (847) 467-1271
Fax: (847) 491-7859
Email: satoru@northwestern.edu

NORTHWESTERN
UNIVERSITY

May 7, 2021

Dear Reviewer 2,

We appreciate your thoughtful and constructive comments. Below we pasted your comments in blue text followed by our response in black text. Bolded red text indicates the sections in the revised manuscript where we addressed your comments. We think that the manuscript has improved as a result of addressing your concerns. Thank you very much again for your help with improving the manuscript.

Best,

Satoru Suzuki

Reviewer #2: The authors performed the spectral analysis of multichannel EEG signals recorded in healthy persons during the resting state. The results of this study and the way they are presented are very interesting and original.

Our response:

We thank the reviewer for their encouraging comments on our manuscript.

However, the assumption that the power spectrum is a measure of brain synchronization is not correct. The phase synchronization methods like Phase Locking Value or Phase Lag Index are used most often for this purpose. Moreover, the authors having the sixty-channel scalp EEG data, were able to perform the source-level spectral analysis.

Our response:

We thank the reviewer for pointing out the fact that we did not clearly explain what we meant by synchronization and desynchronization in the context of the manuscript. PLC and PLI pertain to phase clustering between electrodes. We examined spectral power per electrode (more precisely per site because we surface-Laplacian transformed raw EEG data). Thus, what we measured was the temporal dynamics of spectral power per electrode (site). A typical interpretation is that higher spectral power reflects greater oscillatory synchronization of LFP activity across a large neural population that reaches the particular electrode (site) because non-synchronized activities average out due to massive summation. Nevertheless, EEG spectral power also reflects non-oscillatory components such as the 1/f^b spectral background which may reflect random-walk type neural noise and excitatory/inhibitory balance, and some harmonics from non-sinusoidal oscillatory activities. We include these caveats in the revision. **In the revised manuscript, the relationship between EEG spectral power and oscillatory synchronization of local neural population is discussed in the second paragraph on page 3. Contributions of non-sinusoidal oscillations and non-oscillatory components to EEG spectral power are discussed in the first two paragraphs on page 33.**

With respect to EEG source identification, we used a data-driven approach, which is typically referred to as the surface-Laplacian transform or current-source-density transform. We prefer this method of EEG

source identification to those that generate pseudo brain-activation maps. The reason is that the surface-Laplacian transform provides a reference-free measure, substantially reduces volume-conduction effects, and fairly accurately identifies neural sources near the cortical surface (citations included in the paper) without making convenient/generic assumptions that other “source-modeling” algorithms make to map EEG data with only 16-256 degrees of freedom into “voxels” with massive degrees of freedom (note that LORETA algorithm is based on the computation of current-source densities). Our personal view is that “brain-source-localization” methods not guided by individual participants’ structural MRI generate brain-activation images that are misleading.
In the revised manuscript, a brief rationale for using the surface-Laplacian transform (to infer macroscopic current source/sink densities over the cortical surface) is provided with appropriate citations in the last paragraph on page 6.

There are several incorrect citations in this paper. The expression: “see citations above” should be replaced by appropriate numbers of references. Moreover, the authors should not cite their paper that are under review and it has not yet been published (reference 27).

Our response:

For papers referenced multiple times throughout the manuscript, we now always clarify the citations. The manuscript in question is now published.
Menceloglu, M., Grabowecky, M., & Suzuki, S. (2021). Probabilistic, entropy-maximizing control of large-scale neural synchronization. *PLoS ONE 16(4)*: e0249317.
https://doi.org/10.1371/journal.pone.0249317

More citations are needed in the Introduction section (the last paragraph on the page 3 and the last paragraph of the Introduction section on the page 4). The results described in the Discussion section should be compared with the results of other authors.

Our response:

We agree that our background literature review is unusually general compared with those in most other papers. This is because most studies are motivated by a knowledge gap in the existing literature and the results fill the gap. The introduction and discussion sections of a typical research article therefore closely describe the study’s association with a specific group of prior studies. Our goal was to uncover the rules (if any) that the spatiotemporal dynamics of maximal and minimal spectral power obey. The rules that we discovered were not motivated or anticipated by any prior studies, nor do they particularly influence the interpretations of a specific group of prior studies or theories. The reader may choose to consider incorporating the rules we discovered as a building block into their theoretical and/or experimental work as they see fit.
In the revised manuscript, we tried to clearly articulate, especially in the Introduction section, that we took an observational-science (discovery-oriented) approach for this study. We have also added a discussion (on page 4) of how the particular analysis approach we took in this study is complementary to the typical correlation-matrix based approaches for understanding networks of oscillatory neural activity.

The term ‘1 non-binary’ in the first phrase of ‘Participants’ section is not clear. The symbols M and SD can be replaced by ‘mean age: X ± Y years’.

Our response:

“non-binary” means that the participant declined to identify their gender as either woman or man. We appreciate the reviewer’s suggestion for reformatting participants’ age range, and incorporated it in the methods section.
In the revised manuscript, we now provide an explanation of "Non-binary", and the age formatting recommended by the reviewer is implemented in the second paragraph on page 5.

There are several phrases which should be explained better.
¬ In ‘EEG recording and preprocessing’ section: “To reduce effects of volume conduction (to approximately within adjacent sites; e.g., [32] ???), to virtually eliminate the effects of reference electrode choice (verified ???), as well as to facilitate data-driven determinations of EEG current sources ??? ”;

Our response:

We appreciate the reviewer for pointing out these descriptions that were unclear. We have expanded these descriptions to improve clarity.
In the revised manuscript, we elaborate more on the use of the surface-Laplacian transform in the last paragraph on page 6.

¬ In ‘EEG analysis’ section several expressions are unclear: “macroscopically estimating the underlying electrical currents”; “without having to estimate and discount them in sliding-time windows”; “stochastically (i.e., unpredictably in a memory-free manner)”.

Our response:

We have expanded these descriptions in the revision to improve clarity.
In the revised manuscript, our rationale for taking the temporal derivative of EEG is discussed in the first paragraph on page 7. The relationship between the exponential distribution of phase-scrambled spectral power and stochastic dynamics is described in the second paragraph on page 7.

¬ In ‘Spatial distributions of spectral-power activations and suppressions’ section: “capped at 1/nsites – 1/60”; “floored at -1 (100% below the chance level)”.

Our response:

We apologize for the unclear description. If an isolated activation (occurring at only one site) always occurred at the same site, the probability of occurrence at that site (relative to chance) would be 1–1/60. If twin activation (n=2) always occurred at the same two sites, the probability of occurrence at each of the two sites (relative to chance) would be 1/2–1/60. Similarly, if triple activation (n=3) always occurred at the same three sites, the probability of occurrence at each of the three sites would be 1/3–1/60. The reason is that a spatial probability distribution has to sum to 1. Thus, the ceiling is (1/nsites–1/60)/(1/60)*100 because we define the relative probability as the percent change from the chance level (1/60). The floor occurs when the probability of occurrence is zero, which translates to 100% reduction from the chance level, that is, (0–1/60)/(1/60)*100 = –100. We will clarify these points in the revision.
In the revised manuscript, we tried to more clearly explain the meanings of "cap" and "floor" in the second paragraph on page 13.

The bottom row of Figure 2 with remaining four conditions is redundant because results are similar to that for the first condition (rest with eyes closed). Whereas, two panels in the upper row could be divided in two separated figures.

Our response:

We appreciate this suggestion. We think that it is worthwhile to show the results from all conditions to demonstrate the consistency of the results across all behavioral conditions. The fact that the reviewer felt that the presentation of the results from all conditions would be redundant attests to the strong consistency of the results. Regarding the second point, we prefer that the illustrative examples and the summary plots be shown in the same figure.

The descriptions of Figures, especially Figures 1 and 2, are too long. It should be moved to the main text.

On the page 24 there is an error in the numbering of cited figures (Figures 8-12 should be replaced by Figures 3-7 in the second paragraph).

Our response;

We appreciate these comments. We shortened the captions for Figures 1 and 2. We also revised the captions for the remaining figures to improve clarity. We agree that there are some redundancies between the captions and the main text, but we prefer that each figure be self-contained in that the reader can roughly understand the data presented in the figures without reading the main text. As for the second paragraph on page 24, we actually intended to refer to Figures 8-12 (not Figures 3-7). In that paragraph, we compare the overall patterns averaged across all contexts (Figures 3-7) with the patterns from different contexts (Figures 8-12). We have revised the relevant paragraph to avoid the confusion.
In the revised manuscript, we now clearly state in the first paragraph on page 23 that the spatial distributions of spectral-power activations and suppresions under representative temporal contexts are shown in Figures 8-12. We also shortened the captions for Figures 1 and 2 by 8% and 18%, respectively.
